# Chronic intermittent hypoxia promoted lung cancer stem cell-like properties via enhancing Bach1 expression

**DOI:** 10.1186/s12931-021-01655-6

**Published:** 2021-02-17

**Authors:** Shengyu Hao, Xiaodan Zhu, Zilong Liu, Xiaodan Wu, Shanqun Li, Pan Jiang, Liyan Jiang

**Affiliations:** 1 Department of Pulmonary Medicine, Zhongshan Hospital, Fudan University, 180 Fenglin Road, Shanghai, 200032 China; 2 Clinical Center for Sleep Breathing Disorder and Snoring, Zhongshan Hospital, Fudan University, Shanghai, 200032 China

**Keywords:** Obstructive sleep apnea, Chronic intermittent hypoxia, Cancer stem cells, Lung cancer, Bach1

## Abstract

**Background:**

An adverse role for obstructive sleep apnea (OSA) in cancer aggressiveness and mortality has recently emerged from clinical and animal studies, and the reasons have not been fully determined. Cancer stem cells (CSCs) are regarded as the main cause of carcinoma metastasis. So far, the relationship between OSA and lung CSCs has not been explored.

**Method:**

In the present study, we established an orthotopic mouse model of primary lung cancer and utilized chronic intermittent hypoxia (CIH) exposure to mimic OSA status.

**Results:**

We observed that CIH endows lung cancer with greater metastatic potential, evidenced by increased tumor growth, tumor seeding, and upregulated CSC-related gene expression in the lungs. Notably, the transcription factor BTB and CNC homology 1 (Bach1), a key factor in responding to conditions of oxidative stress, is increased in lung cancer after CIH exposure in vitro and in vivo. Meanwhile, exposing lung cancer cells to CIH promoted cell proliferation, clonal diversity, induced stem-like cell marker expression, and gave rise to CSCs at a relatively higher frequency. Furthermore, the increase of mitochondrial ROS (mtROS) and CSC-marker expression induced by CIH exposure was abolished in *Bach1* shRNA-treated lung cancer cells.

**Conclusions:**

Our results indicated that CIH promoted lung CSC-like properties by activating mtROS, which was partially mediated by Bach1.

## Background

Obstructive sleep apnea (OSA) is a highly prevalent disorder characterized by repetitive occlusion of the upper airway during sleep that results in chronic intermittent hypoxia (CIH) [1]. OSA is associated with a wide array of morbidities and mortalities including metabolic syndrome, cardiovascular and cognitive disorders [2]. Besides, accumulating evidence of animal models and clinical researches has revealed that OSA exacerbated tumor progression and metastasis, which have been reported in breast cancer [3], colorectal cancer, prostate cancer, melanoma [4, 5], renal carcinoma [6], and lung cancer [7, 8]. Notably, a recently published study prospectively provided evidence that about 50% of lung cancer patients have moderate to severe OSA. The relationship between OSA and lung cancer has been explored in recent years. However, the underlying mechanism for CIH deteriorating tumor remains obscure.

Lung cancer is on rising globally and is the main cause of cancer-related death worldwide [9]. The cancer stem cells (CSCs) involved in the lung cancer process have been explored and well established [10]. CSCs are described as a subpopulation of tumor cells with the capacity to self-renew, differentiate, and promote tumor growth. They are responsible for metastases development and recurrence after therapy [11, 12]. Lung CSCs have been previously isolated based on the presence of cell surface markers, including CD133 and CD44 [13, 14]. Hypoxia is increasingly recognized as owing the potential to exert a significant effect on the maintenance and evolution of CSCs [15, 16]. In contrast to continuous hypoxia, CIH in patients with OSA is a unique physiological state with a phase of post-hypoxic re-oxygenation. It is characterized by higher frequency, more serious hypoxia, and larger variation in blood oxygen saturation. However, little is known about the effect of OSA-like CIH on CSCs and the underlying mechanisms.

Reactive oxygen species (ROS), especially mitochondrial ROS (mtROS), is considered as a core factor in the pathogenesis of OSA-related diseases [16]. Though many researchers have demonstrated the connection between ROS production and CSCs, the role of ROS in cancer initiation and progression is controversial. Cancer cells can generate high levels of ROS [17]. Although ROS is often beneficial for tumor cells, specific forms of ROS can stimulate pro-tumorigenic signaling pathways by oxidizing and inactivating tumor suppressor proteins [18, 19]. Bach1 is considered as a key factor in responding to conditions of oxidative stress and is associated with poor survival and metastasis in lung cancer patients [20]. Furthermore, we found that Bach1 can promote lung cancer stem cell phenotypes in our previous research (has not been published).

However, the effects of CIH on CSCs and its possible role in tumor metastatic phenotype are unknown and the role of Bach1 plays in CIH-promoted lung cancer is unclear. Based on previous studies, we hypothesized that the adverse effects of CIH on tumor proliferation and invasion would be partly mediated via CSCs. Our findings uncover that CIH plays a role in promoting CSC-like property, in which Bach1 and mtROS are activated. Our study suggests the possibility of developing CSC-targeting agents for lung cancer treatment in OSA patients.

## Materials and methods

### Experimental animals

**Fig. 1:**
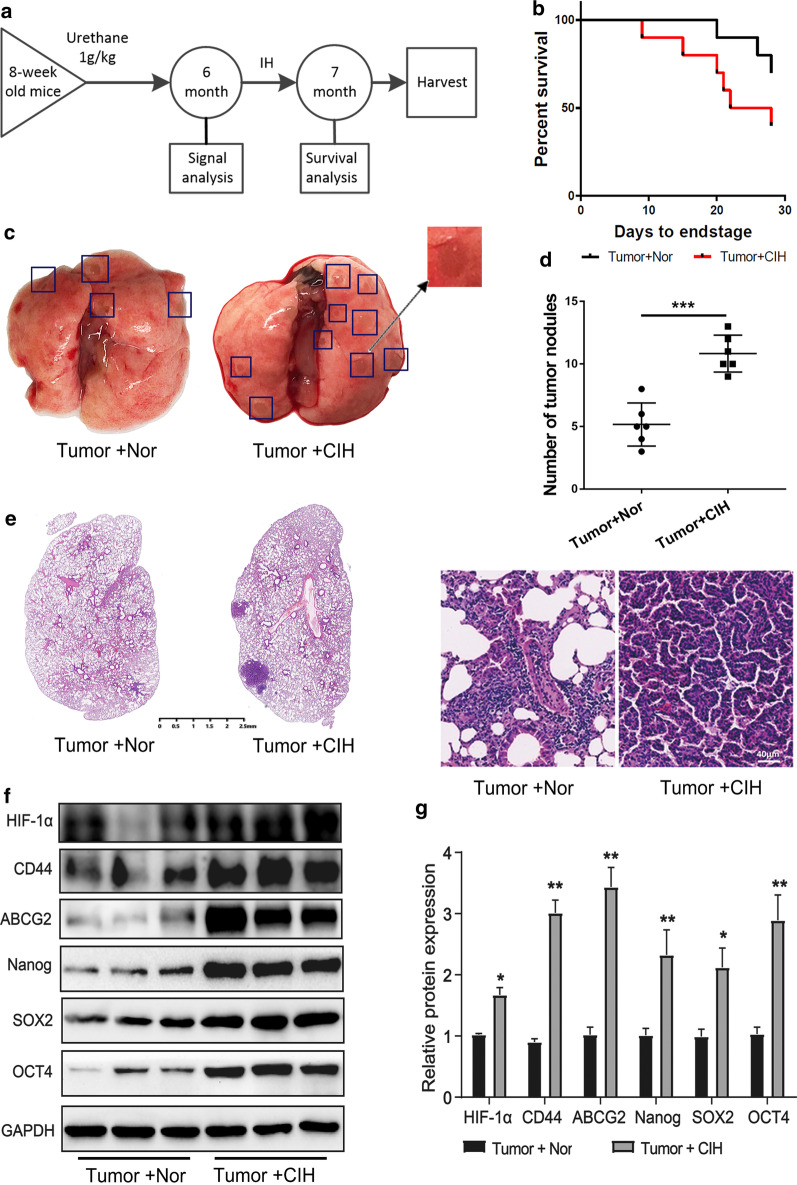
Lung cancer in CIH-treated mice has greater CSC-like potential. **a** The timeline of this experiment. Briefly, after injected with urethane, mice were cultured for 6 months, then exposed to CIH for 1 month. All mice were harvest at the end of the experiment. **b** Survival rates of C57Bl/6 wild-type male mice. Tumor + Nor, n = 12, Tumor + CIH, n = 12. **c** The representative images of cancer loci in lungs from Tumor + Nor and Tumor + CIH groups. **d** The number of cancer loci observed in the lungs of mice. Data are shown as mean ± SEM. **e** Images of lung cancer nodules in H&E-stained lungs of mice. (**f** and **g**) The protein levels of HIF-1α, CD44, ABCG2, Nanog, SOX2, and Oct4 in the lung cancer nodules were detected by western blot analysis. GADPH expression served as a loading control. Data are shown as mean ± SEM. Error bars represent the mean. *P < 0.05, **P < 0.01 and ***P < 0.001. *CIH* chronic intermittent hypoxia, *Nor* normoxia, *CSC* cancer stem cell

C57BL/6 male mice of 8 weeks of age were purchased from the Nanjing Model Animal Center and bred in the animal facility of Zhongshan Hospital Fudan University according to the National Institutes of Health Guidelines for the Humane Treatment of Laboratory Animals. All procedures involving mice were approved by Zhongshan Hospital Institutional Animal Care and Use Committee by the Helsinki Declaration of 1975. Mice were treated with urethane (1 g/kg, Sigma, St. Louis, MO, USA) in total 0.2 ml PBS by intraperitoneal injection with a gap of 48 h in a week for 6 weeks. At 6 months after urethane injection, the mice were randomly assigned to two groups (n = 12 per group): (I) Tumor + Nor group: mice in tumor group cultured under normoxic conditions; (II) Tumor + CIH group: mice in tumor group exposed to CIH. The model of CIH was established according to our previous research [21]. Briefly, mice were exposed to 1-min period of CIH cycle in a chamber, and the oxygen concentration was adjusted between 4 and 21%. Nitrogen was delivered to the chambers at a rate sufficient to achieve a fraction of inspired oxygen (FiO2) of 4–7% within 30 s and maintain this level of FiO_2_ for 10 s. Then, oxygen was introduced to achieve FiO2 of 20–21% within 30 s. This model mimics a rate of 60 apneas/h^−1^, which is typical of severe OSA and performed for 8 h/day (8:00–16:00) for 1 month. The timeline was shown in Fig. 1a. After 8 h at the end of the last CIH cycle, mice were immediately sacrificed, and lung lobes were carefully separated from the mice. The number of carcinoma colonies per lung was counted after anterior and posterior image acquisition.

### Cell culture and CIH modeling in vitro

A549 and SPCA1 cells were purchased from the Chinese Academy of Sciences Shanghai Cell Bank and cultured in RPMI-1640 supplemented with 10% fetal bovine serum in a humidified atmosphere containing 5% CO_2_ at 37℃. The medium was changed at 2-day intervals and cells re-plated at 80–90% confluence. CIH or normoxia model in vitro was performed as our previous study [22]. Briefly, cells were cultured in a computer‐controlled incubator chamber attached to an external O_2_–CO_2_ computer‐driven servo controller (Biospherix, Lacona, NY), where the O_2_ concentration was altered between 0 and 21% every 30 min by injecting N_2_ or O_2_ with 5% CO_2_. The dissolved O_2_ inside the culture medium was monitored by a laser O_2_ probe (Biospherix) and the CIH reached 5% O_2_ and 21% O_2_ as hypoxic and normoxic values according to the sensing of the cells. Normal air conditions corresponded to 21% O_2_ and 5% CO_2_.Human *Bach1* or control (Genechem, Shanghai, China) shRNA were transfected into A549 and SPC cells with Lipofectamine 2000 (Invitrogen) according to the manufacturer's instructions.

### Immunohistochemistry

After flushing, the lower lobe of each lung was stored at − 80 °C for RNA and proteomic analyses. The upper lobe of the lung was fixed by perfusion with 3.8% paraformaldehyde and embedded in paraffin. Tissue sections (4 μm) were stained with hematoxylin and eosin (HE) for routine histological analysis.

### Cell viability and Edu assy

Cells were plated onto 96-well plates in triplicate at a density of 1 × 10^3^ cells per well and allowed to adhere overnight in 1640 medium. Cells were cultured under Nor or CIH conditions for different time points. Before detection, 10 μM/well of CCK8 was added and incubated for 2 h. The absorbance was measured at 570 nm using a microplate spectrophotometer. Edu (BeyoClick™ EdU, Beyotime) staining was used and performed following the manufacturer's instructions for labeling of the nucleus of dividing cells. The cells were treated with 10 μM EdU. After 2 h, the cells were fixed and incubated with DAPI. Then, the cells were observed on a laser scanning microscope.

### Colony formation assay

A549 and SPCA1 were seeded in six-well plates at a density of 800 cells/well. Cells were cultured under Nor or CIH conditions. Two weeks later, cell colonies were fixed and stained with crystal violet. A colony was defined as 50 cells.

### Flow cytometric analysis

A549 and SPCA1 were seeded in 12-well plates at a proper concentration and cultured under Nor or CIH condition for 48 h. At the end of CIH cycles, cells were harvested, filtration and centrifugation, and FITC-labeled anti-CD44 (555478) and APC-labeled anti-CD133 (53276) (Cell Signaling Technologies, USA) were used for surface staining. The adherent cells were treated with mitoSOX™ red mitochondrial superoxide indicator (Invitrogen™) at a final concentration of 5 μM for 10 min at 37 °C and washed with PBS three times. Then the cells were harvested, filtration and centrifugation. Cells were detected by the quantitation of fluorescence intensity by flow cytometry. All data were analyzed with FlowJo software (Tree Star Inc., San Carlos, CA).

### Sphere formation assay

Cells were indicated with 10 ng/mL of human recombinant basic fibroblast growth factor (R&D Systems, Minneapolis, MN) and 20 ng/mL of epidermal growth factor (R&D Systems) in serum-free Dulbecco's Modified Eagle's Medium-F12 (Gibco) medium. Spheres were observed using a microscope (Olympus, Tokyo, Japan).

### Western blot analysis and quantitative real-time PCR

Cell and tissue lysates were prepared and performed as previously described. Briefly, membranes were blocked with 5% nonfat dry milk in TBST for 1 h and incubated overnight at 4 °C with primary Abs against CD133 (64326S), CD44 (37259S), Oct-4 (2750S), Nanog (4903S), Sox2 (14962S), ABCG2 (42078S), Bach1 (4578), HIF-1α (14179), and GADPH (5174) (purchased from Cell Signaling Technologies). The membranes were incubated with an anti-rabbit horseradish peroxidase-conjugated secondary Ab (Cell Signaling Technologies), and protein bands were detected by chemiluminescence using Immobilon Forte Western HRP substrate (Millipore WBLUF0500). Total RNA was extracted from A549 or SPCA1 using TRIzol reagent (Takara Bio, Shiga, Japan). cDNA was synthesized from the isolated RNA, and quantitative PCR was performed according to the manufacturer's instructions. This quantitative assay was performed using an SYBR QPCR kit (Toyobo, Osaka, Japan). The primer sequences used for PCR were provided in Table 1. Densitometry was performed using ImageJ software (NIH, Bethesda, MD). All experiments were performed in triplicate.

**Table 1:** Primers used for real-time PCR

Genes	Forward (5′-3′)	Reverse (5′-3′)
GAPDH	GCACCGTCAAGGCTGAGAAC	TGGTGAAGACGCCAGTGGA
CD44 Sox2 Nanog Oct4 CD133	GCATTGCAGTCAACAGTCGAAGA GACAGTTACGCGCACATGAA GTGATTTGTGGGCCTGA AGA GGTATTCAGCCAAACGA CCA GAACAGGGCTACTCGCAAAG	CCTTGTTCACCAAATGCACCA TAGGTCTG CGAGCTGGTCAT ACACAGCTGGGTGGAAGAGA CACACTCGGACCACATCCTT AAAGG GCAGTTGACGGAAC

### Statistical analyses

For in vitro experiments, data represent at least three biological replicates performed with a minimum of duplicate technical replicates unless indicated otherwise. The two-tailed, unpaired Student's t-test was used to determine statistical significance for all experiments unless indicated otherwise. Statistical significance was denoted as follows: *P < 0.05, **P < 0.01, ***P < 0.001, ****P < 0.0001. Data are shown as mean ± SEM unless indicated otherwise.

## Results

**Fig. 2:**
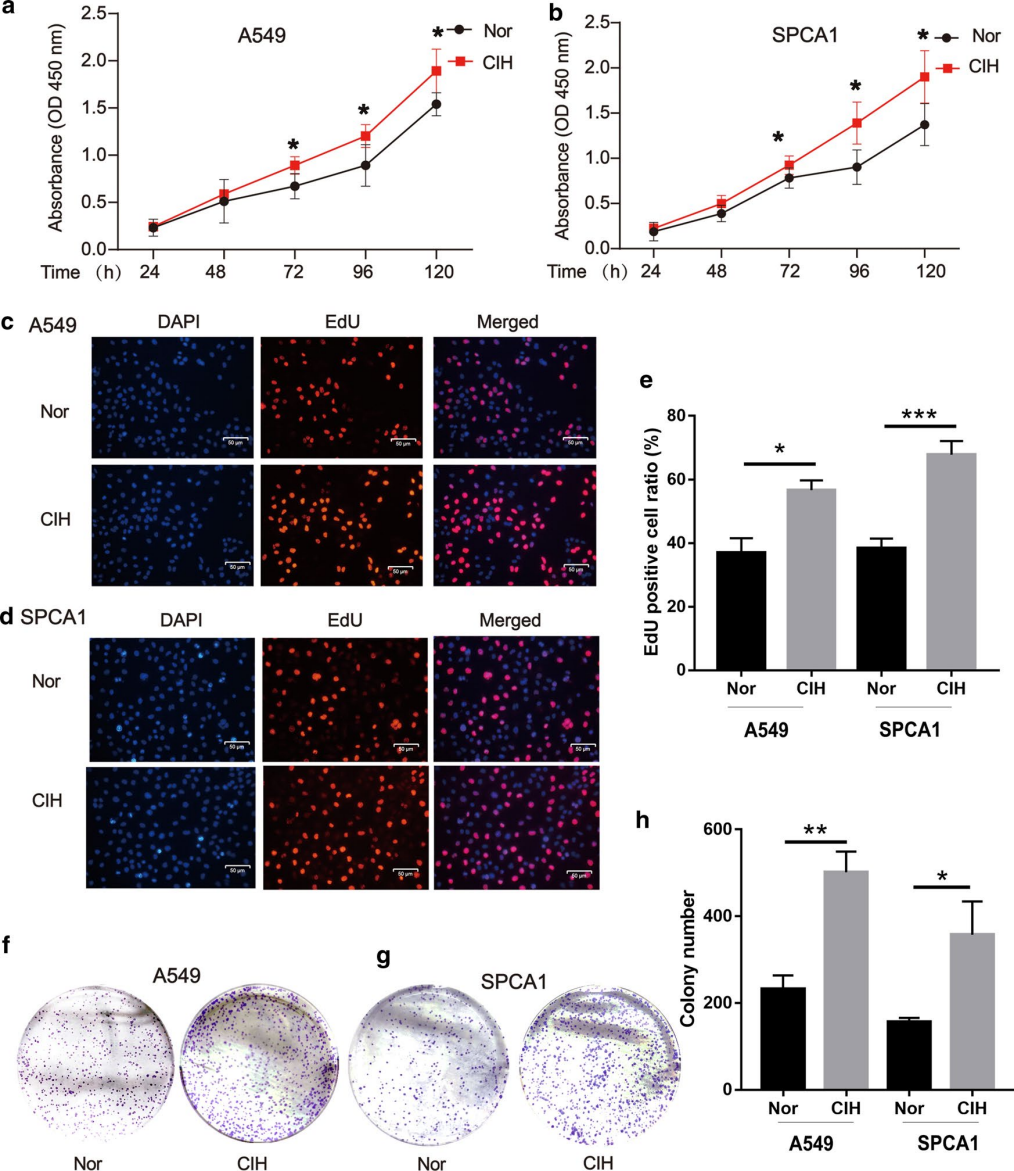
CIH promoted lung cancer cell proliferation. Cell validations of A549 (**a**) and SPCA1 (**b**) were detected by CCK-8 assay after exposure to Nor or CIH for 24, 48, 72, and 96 h. Cell proliferation of A549 (**c**) or SPCA1 (**d**) after 48-h Nor or CIH exposure was assessed using Edu assay. **e** The percentage of Edu positive cells in A549 or SPCA1 was analyzed and illustrated. **f**, **g** Assessment of cancer genericity was performed by colony formation analysis after CIH treatment. **h** The number of colonies was measured and illustrated. Error bars represent the mean ± SEM of at least triplicate experiments. *P < 0.05, **P < 0.01. *CIH* chronic intermittent hypoxia

**Fig. 3:**
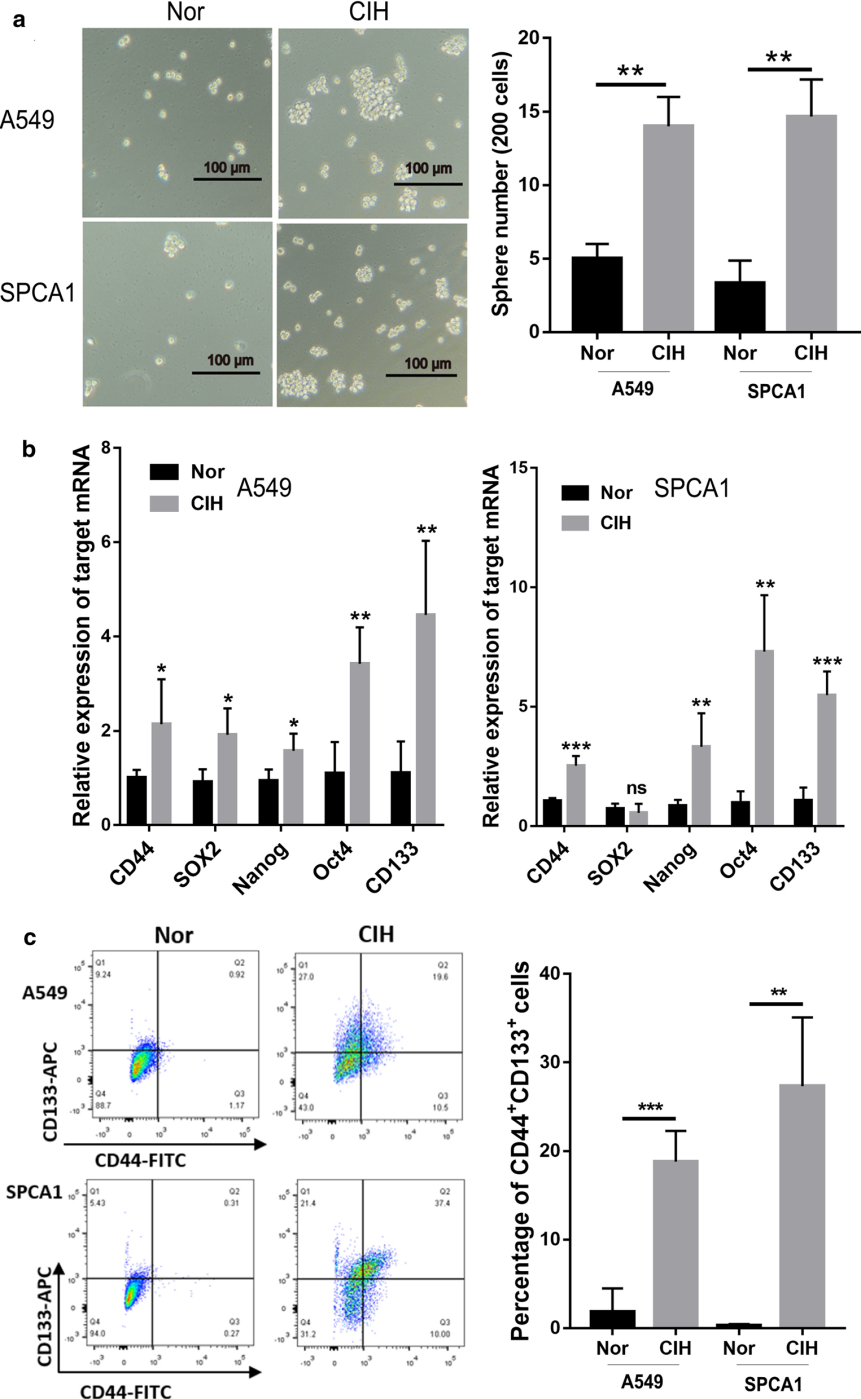
CIH promoted CSC-like properties in NSCLC cells. **a** Microscopic observation of NSCLC cells. A549 and SPCA1 spheroids were obtained and then cultured under Nor or CIH conditions for 24 h. **b** qRCP analyses in triplicate of CD44, Sox2, Nanog, Oct4, and CD133 in NSCLC cells. Adherent A549 and SPCA1 were treated with 24 h-Nor or CIH exposure. GADPH expression served as an internal control. **c** Flow cytometry analyses of the percentage of CD44^+^CD133^+^ cells in A549 or SPCA1 populations. Error bars represent the mean ± SEM of at least triplicate experiments. *P < 0.05, **P < 0.01. *CIH* chronic intermittent hypoxia, *CSC* cancer stem cell

**Fig. 4:**
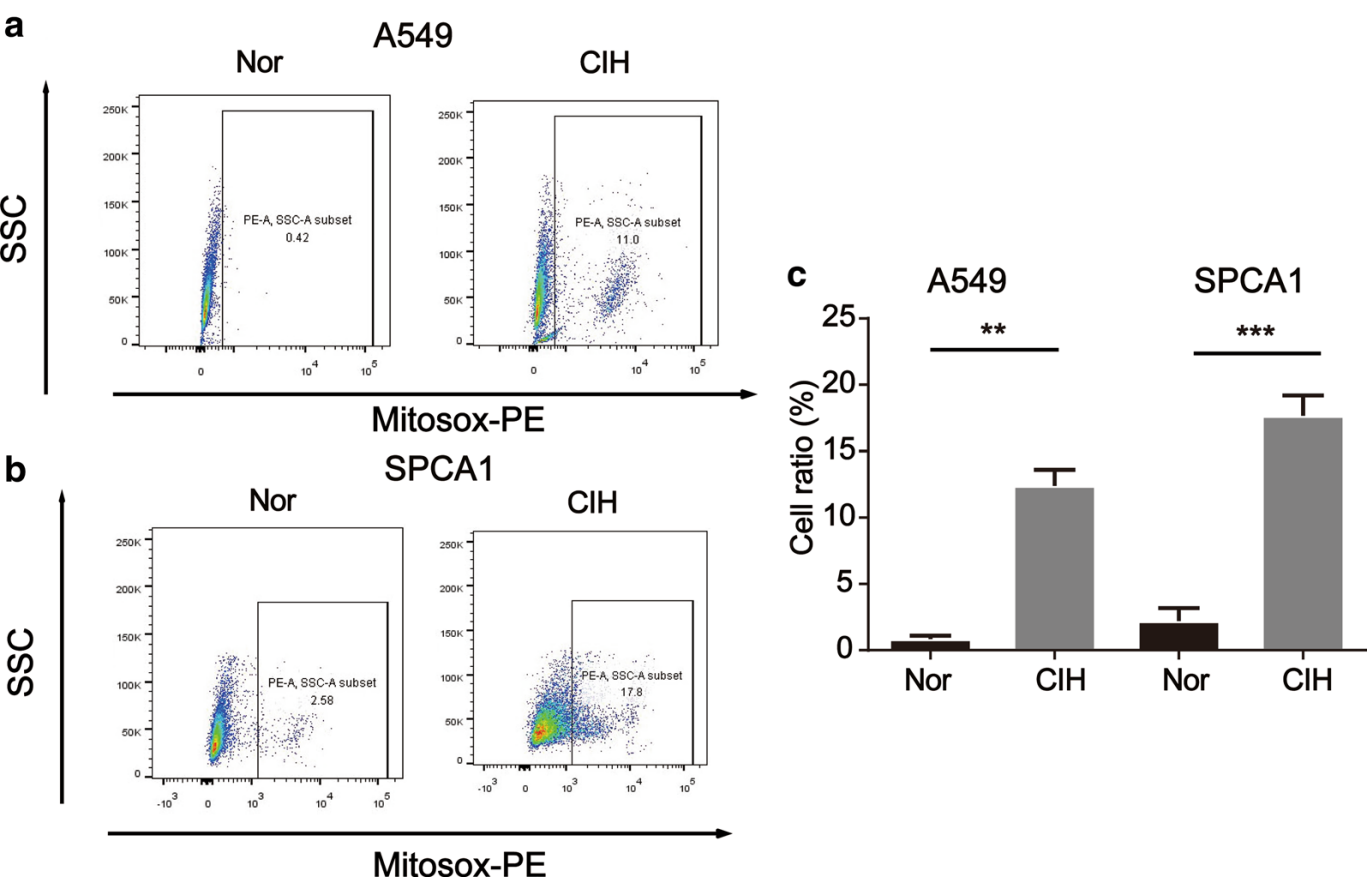
CIH induces mitochondrial ROS accumulation in NSCLC cells. Flow cytometric analysis of A549 (**a**) or SPCA1 (**b**) treated with MitoSOX after CIH exposure for 48hrs is shown. **c** Quantitative analysis of MitoSOX-positive cells is shown. All data are presented as the mean ± SEM. *P < 0.05, **P < 0.01 and ***P < 0.001 compared with the control group. Every experiment was repeated at least three times. *CIH* chronic intermittent hypoxia

**Fig. 5:**
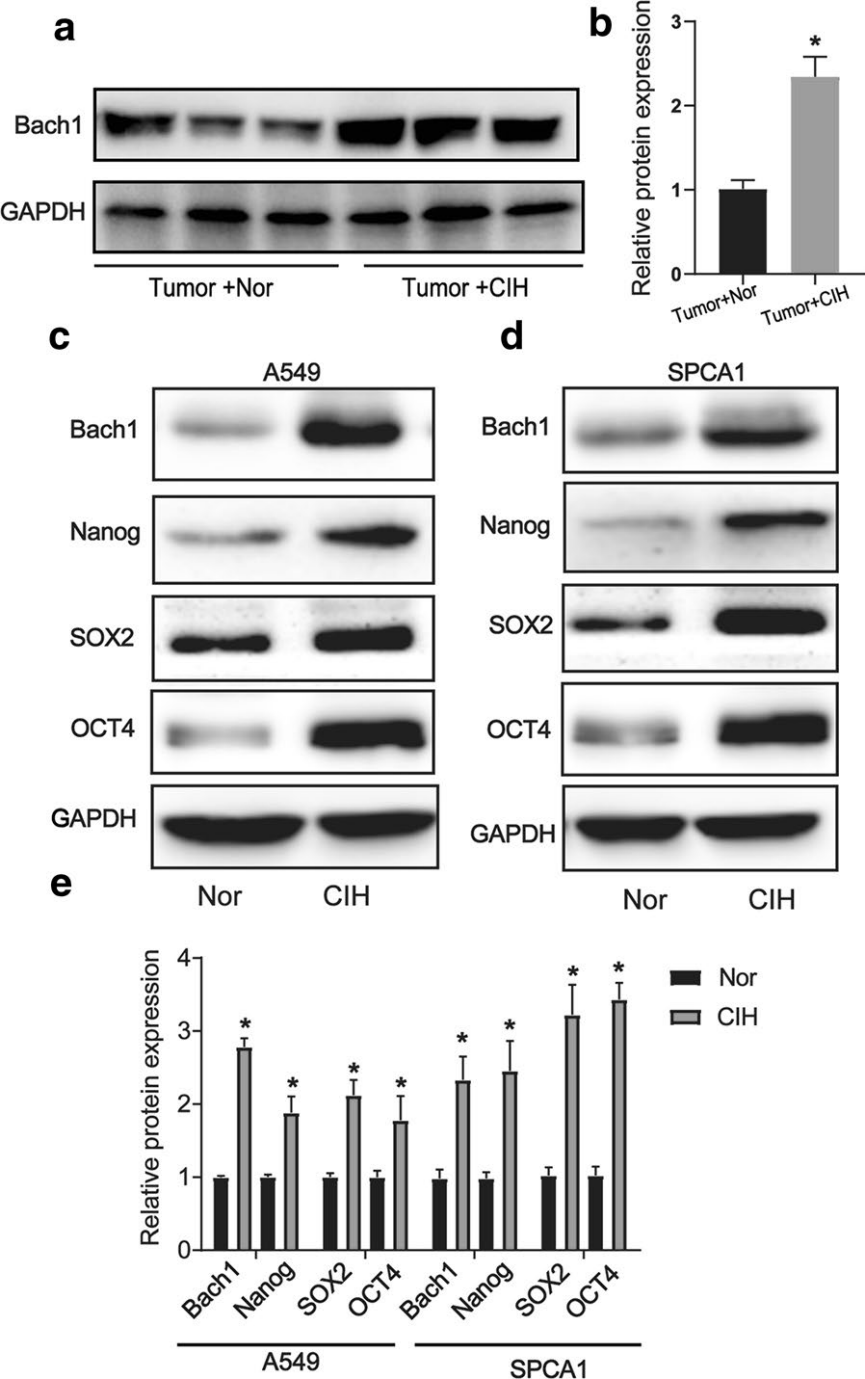
CIH increased Bach1 expression and CSC-like property. **a** The protein expression of Bach1 in lung cancer nodules from Tumor + Nor or Tumor + CIH mice by Western blot analysis. **b** Quantification of relative protein expression was performed by densitometric analysis, and GADPH acted as an internal control. The protein expression of Bach1, Nanog, SOX2, and OCT4 in A549 (**c**) or SPCA1 (**d**) after CIH exposure for 48 h by Western blot analysis. **e** Quantification of relative protein expression was performed by densitometric analysis, and GADPH acted as an internal control. All data are presented as the mean ± SEM. *P < 0.05 compared with the control group. Every experiment was repeated at least three times. *CIH* chronic intermittent hypoxia, *CSC* cancer stem cell

1. **CIH promoted carcinogen-induced Tumor growth**
  To explore the effect of CIH on lung carcinogenesis, an orthothopic lung tumor model was established in WT mice by induction with a cumulative high dose of urethane injection. After six months, the mice were randomly cultured under normoxia (Nor) or CIH condition as we described in Methods (Fig. 1a). We observed that Tumor + CIH groups exhibited lower survival rates and accelerated tumor growth than Tumor + Nor groups (Fig. 1b). The lungs of mice in the Tumor + CIH group contained a greater number and larger metastases compared to Tumor + Nor group (Fig. 1c–e). CD44, ABCG2, Nanog, SOX2, and Oct4 were identified as important stem-cell transcription factors or markers involved in maintaining the CSC-like phenotypes in many kinds of cancer. To verify the influence of CIH on CSC-like properties of lung cancer, the protein levels of these markers were detected by western blot tests in cancer nodules from Tumor + Nor and Tumor + CIH groups. As shown in Fig. 1f and g, the expression levels of CD44, SOX2, Nanog, Oct4, and ABCG2 in lung cancer nodules from Tumor + CIH groups were significantly upregulated compared with the lung cancer tissues from Tumor + Nor mice. The above results indicated that CIH promoted stemness in lung cancer.
2. **CIH promoted lung cancer cell proliferation**
  Next, we employed A549 and SPCA1, two commonly used non-small cell lung cancer (NSCLC) lines to further validate the effect of CIH on the proliferation of NSCLC cells. A549 or SPCA1 were cultured under CIH or Nor conditions for 24, 48, 72, 96 h, and the proliferation of cells was tested by the CCK-8 assay at different time points. As presented by optical density, A549 or SPCA1 cultured under CIH conditions exhibited significantly increased cell validation compared with the cells cultured under Nor conditions (Fig. 2a and b). Additionally, CIH exposure statistically increased the percentage of Edu-positive cells (Fig. 2c–e). The colony-forming ability of cells was also enhanced by CIH exposure validated by plate cloning test (Fig. 2f–h).
3. **CIH induced CSC-like phenotypes in lung cancer cells**
  To further determine the effect of CIH on the formation of CSCs in NSCLCs, a sphere-forming assay was performed to detect the capacity of CSC-like cell self-renewal in our study (Fig. 3a). We found that CIH promoted the formation of spheres. Meanwhile, CD44, CD133, Nanog, Oct4, and SOX2 mRNA were increased in CIH-treatment NSCLC cells validated by RT-PCR (Fig. 3b). As CD133 and CD44 are two important CSC surface markers in several types of cancer, CIH exposure significantly enhanced the percentage of CD44^+^CD133^+^ subsets in NSCLC cells evidenced by flowmetry analysis (Fig. 3c).
4. **CIH promoted mtROS accumulation and Bach1 expression in vitro and in vivo**
  Many studies have identified ROS as an important regulator in CSCs, and mtROS is a major source of ROS. Though the role of ROS in the cancer process is controversial, we found that CIH notably promoted the levels of mtROS in NSCLC cells in our study (Fig. 4a–c). Bach1 is a transcriptor repressing the expression of the antioxidant enzyme. We previously demonstrated that Bach1 induced CD44 expression to promote CSC-like properties in NSCLCs (have not been published). To further evaluate the role of Bach1 in OSA-deteriorated lung cancers, we compared the expression levels of Bach1 in lung cancer nodules and NSCLC cells between CIH and Nor conditions. As shown in Fig. 5a and b, Bach1 was obviously increased in lung cancer nodules from Tumor + CIH mice compared with those from Tumor + Nor mice. Furthermore, CIH-treated A549 and SPCA1 presented higher expression levels of Bach1 than Nor treated cells. Nanog, SOX2, and OCT4 were also determined in CIH and Nor treated cells, and the same tendency was observed (Fig. 5c–e).
5. **Bach1 knockdown attenuated CIH-induced mtROS accumulation and CSC-like phenotypes**

**Fig. 6:**
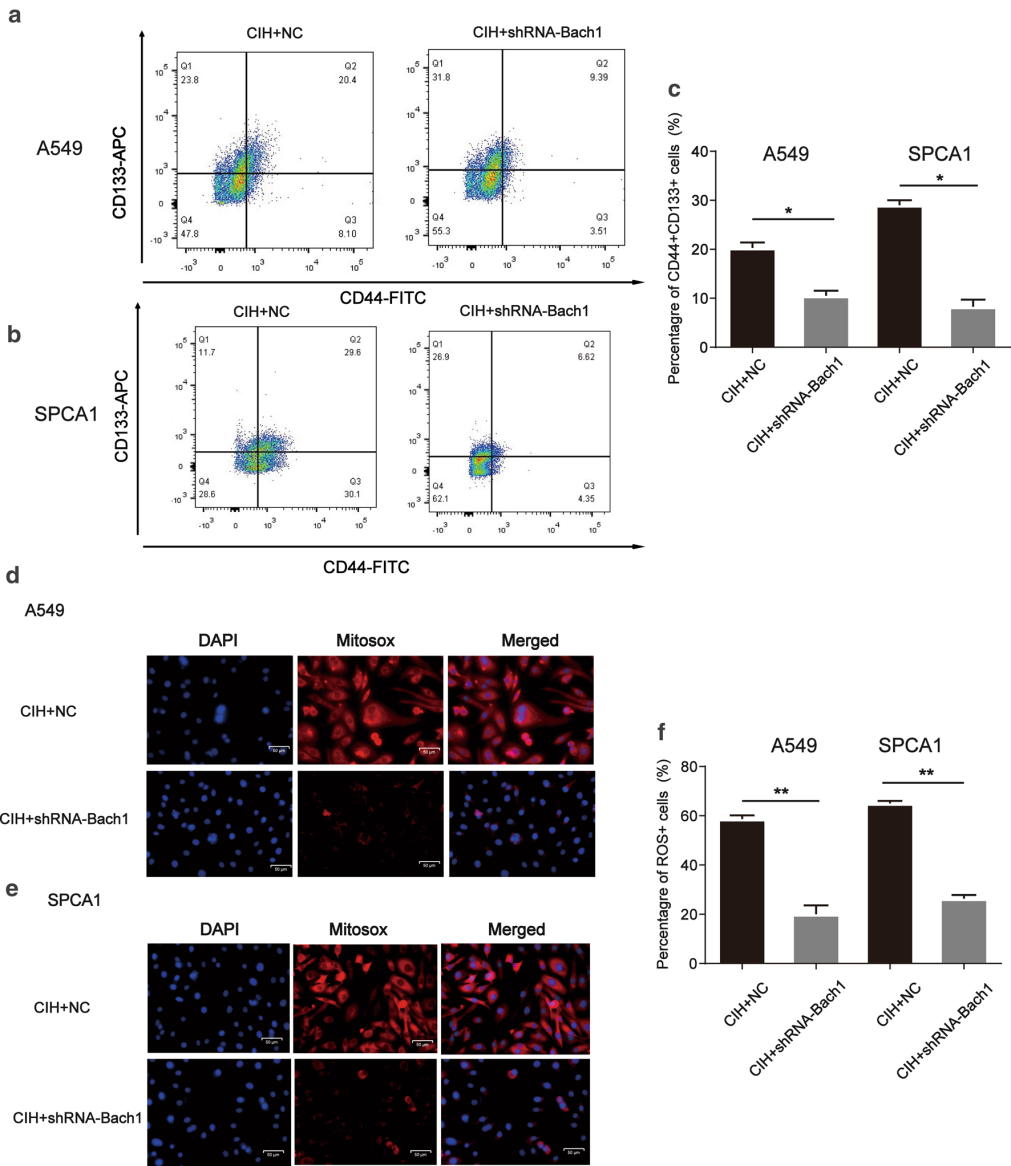
Knockdown of Bach1 decreased the stemness and mitochondrial ROS accumulation in CIH-treated NSCLCs. *Bach1* shRNA or parental negative control (NC) was transfected into A549 or SPCA1. Then the cells were cultured under CIH conditions for 48hrs. Flow cytometry analyses of CD44^+^ CD133^+^ cells in Nor or CIH-treated A549 (**a**) or SPCA1 (**b**). **c** The percentage of CD44^+^ CD133^+^ cells in Nor or CIH-treated cells was measured and illustrated. Fluorescence microscopy analysis for localization of mtROS production in A549 (**d**) and SPCA1 (**e**). Nuclei was stained with Hoechst (blue), mitochondria ROS were stained with MitoSOX-red. The merged panels showed the MitoSOX-red positive cells in total cells. **f** The percentage of MitoSOX positive cells was measured and illustrated. All experiments were performed in triplicate, and data are presented as mean ± SEM. *P < 0.05, **P < 0.01 compared with the control group

To further determine the role of Bach1 in CIH-deteriorated lung cancer, *Bach1* shRNA was used in the following experiments. After treated with *Bach1* shRNA, the effect of CIH on CSC-like phenotype induction in NSCLC cells was partly abolished evidenced by a decreased percentage of CD44^+^CD133^+^ A549 or SPCA1 in total cells (Fig. 6a–c). Meanwhile, CIH also failed to further induce mtROS accumulation in NSCLC cells co-treated with *Bach1* shRNA (Fig. 6d–f). Our collective data supported the requirement of Bach1 for mtROS accumulation and the maintenance of CSC property in NSCLCs.

## Discussion

OSA is increasingly recognized as an important promotor in the process of lung carcinoma. In this study, our data show that murine lung cancer exposed to CIH exhibit increased tumor growth. The more deteriorated lung cancer associated with CIH can be partly explained by mtROS accumulation and increased Bach1 expression, resulting in enhanced CSC-like property. To our knowledge, this is the first study uncovering the relationship between OSA and lung CSCs, as well as the possible mechanism for Bach1 involved in CIH-deteriorating lung cancer. Our findings provide a useful model and biological hypothesis to the adverse cancer outcomes reported in lung cancer patients with OSA.

Data from the current study demonstrated for the first time that CIH induces CSC-like property in lung cancer. Despite advances in anti-cancer therapies such as targeted therapies and immunotherapy, lung cancer is still the world's leading cause of cancer mortality with about a 15% five-year survival rate. Furthermore, an increased risk of death and notably reduction of progression-free survival (PFS) were observed in lung cancer patients with severe OSA [23, 24]. Tumor resistance reduces the efficacy of current therapies resulting in relapse, progression, and subsequent patients' death [25]. CSCs referred to as tumor-initiating cells, are increasingly recognized as a key factor in tumor progression, metastasis, and drug resistance [13, 15]. In this study, we established an orthotopic mouse model of primary lung cancer and cultured the mice under CIH conditions to mimic the process in the comorbidity of primary lung cancer and OSA. In line with previous studies [26], we observed that CIH accelerated the development of lung cancer. Additionally, CIH promoted CSC-like properties in vitro and in vivo. Considering the role of CSCs in lung cancer, our research may partly explain the adverse effect of OSA on lung cancer. However, further clinical researches are still needed to verify whether tumor resistance and poor treatment are directly related to OSA.

The possible underlying mechanisms that OSA-like CIH results in facilitating the development and progression of tumors have been proposed in several investigations. Immune response [26, 27] and hypoxia-related factors such as HIF-1α and ROS [23] were the most commonly explored mechanisms in these studies. Bach1 referred to as a transcriptional factor, plays a regulatory role in ROS by inhibiting the transcription of ROS-related genes, such as heme oxygenase-1 (HO-1) [28] and NADPH quinone oxidoreductase 1 (NQO1) [29]. Moreover, Bach1 was also involved in cell cycle [30], immunity [31], and has been shown to suppress angiogenesis [32] and promoted cancer metastasis, such as breast [33], ovarian [34], and lung cancer[35]. In this study, we found that the expression level of Bach1 was increased in lung cancer nodules from CIH-treated mice and NSCLC cells exposed to CIH conditions accompanied by an increased mtROS. Moreover, Bach1 knockdown decreased the expression of mtROS. Growing evidence has also shown that inhibition of Bach1 alleviates oxidative stress [30]. Based on these previous studies, we found that CIH can accelerate mtROS production by promoting Bach1 expression.

Our results also indicated that the increased expression of Bach1 induced by CIH exposure was accompanied by increased CSC-related translators such as Nanog, SOX2, ABCG2, Oct4, and markers including CD44 and CD133. Furthermore, the increased CSC-like properties induced by CIH were repressed by Bach1 knockdown. Though the effects of Bach1 on maintaining CSC-like property were little reported, our observation is consistent with previous reports that Bach1 can regulate self-renewal and stabilize Nanog, SOX2, and Oct4 in human embryonic stem cells [36]. Additionally, our previous study found that Bach1 can promote lung CSC phenotype by inducing CD44 expression (have not been published). This study has some limitations: there lacks clinical evidence that lung cancer tissues from NSCLC patients with OSA present a higher Bach1 expression and lung CSC properties than those from NSCLC patients without OSA. Also, the regulatory mechanism of Bach1 on stemness in lung CSC still needs further research.

## Conclusions

In conclusion, our findings present a promotor role for CIH in lung cancer development. Besides, the results uncover that CIH induces the lung CSC-like properties in a Bach1-dependent way. These observations suggest that Bach1 may serve as a unique strategy for patients with OSA-related lung cancer, but more work is needed to fully verify the role of Bach1 in OSA-deteriorated lung cancer.

## Data Availability

The data that support the findings of this study are available from the corresponding author upon reasonable request.

## Abbreviations

OSA: Obstructive sleep apnea
CSCs: Cancer stem cells
CIH: Chronic intermittent hypoxia
Bach1: Transcription factor BTB and CNC homology 1
mtROS: Mitochondrial ROS
HE: Hematoxylin and eosin
Nor: Normoxia
NSCLC: Non-small cell lung cancer
HO-1: Heme oxygenase-1
NQO1: NADPH quinone oxidoreductase 1
